# Implicit Teacher Theories Regarding the Argumentative Commentary of Multimodal Texts in the Teaching of Spanish as a Native and Foreign Language

**DOI:** 10.3389/fpsyg.2021.749426

**Published:** 2021-10-27

**Authors:** María Teresa Caro Valverde, José Manuel de Amo Sánchez-Fortún, Juana Celia Domínguez-Oller

**Affiliations:** ^1^Teaching Department of Language and Literature (Spanish, English and French), University of Murcia, Murcia, Spain; ^2^Department of Education, University of Almería, Almería, Spain

**Keywords:** argumentation, text commentary, teachers' beliefs, multimodal texts, teaching Spanish, Spanish foreign language

## Abstract

The research works linked to the thinking of the teaching staff influence the relevant influence that implicit theories exert on decision-making about classroom practise and on the academic performance of students. In this sense, the present study focuses on the teaching belief system about the development of argumentation in the commentary of multimodal texts. For this, a quantitative methodology based on non-experimental or ex post facto design with semi-structured and closed survey-questionnaire-type instruments has been selected. From a target population made up of Spanish teachers, 502 respondents selected using the non-probabilistic sampling technique applied the accessibility criterion. An *ad hoc* questionnaire has been drawn up consisting of 28 items digitised electronically using the survey platform of the University of Murcia. It has been structured in two blocks: the first aimed at establishing the sociodemographic and professional profile of the participants and the second at collecting data related to the teachers' beliefs regarding the work of the text commentary in class. The results show five professional profiles defined based on the implicit theories and the pedagogical model to which they are associated. It is also found that the majority declare that they align themselves with non-conservative didactic trends or approaches, centred on the student body and oriented toward the construction of critical knowledge. In this regard, manifest contradictions are detected between his implicit and explicit epistemological convictions. The findings of this study offer guidelines for the design of an effective and efficient argumentative text commentary formative proposal.

## Introduction

### The Paradigm of Teacher Thinking as a Key Element in Improving Education

The study of education improvement actions based on the paradigm of teacher thinking calls for an examination of their thoughts on learning and teaching, as such thinking has an influence on their day-to-day teaching decisions, both of a normal or innovative nature (Clark and Peterson, 1990; Schön, 1998). Its analysis is just as enriching as it is complicated, portraying the systematic structure of the professional background insofar as the explicit and implicit beliefs, values, ideas, and concepts involved in their teaching action. Given such cognitive polyhedron, its extensive investigative spectrum is based on key terms that can be grouped together in two major blocks of study aimed at discovering “the unique characteristics of the practical thinking of every teacher” (Pérez and Gimeno, 1988, p. 61). One block entails the corresponding constructs and conceptions, while the other the implicit theories and beliefs.

The construct (Kelly, 1955) is shaped by individuals with hypotheses and interpretations that afford them a predictive kind of reflective discernment with which to organise and contrast their progressive experience, in an adaptive manner, with the actions and situations entailed in the process itself, all of which is of particular interest in studying education agents through grid techniques (Pope and Ken, 1981). Today they are usually recognised under the term “concepts” (Pratt, 1992), which, in the education field, affect teaching and learning with positions that oscillate between the mere transfer of knowledge, up to the negotiation of meanings, and the fostering of knowledge generation (Hernández-Pina and Maquilón-Sánchez, 2011, p. 169).

Implicit beliefs or theories refer to the personal frameworks that serve as a starting point for making prioritised decisions. Belief may be understood in many different ways: as a conviction or subjective truth that is different from knowledge as an objective truth (Bunge, 2009); as a socially assumed reflective judgment (Ortega Gasset, 1976); and, as a psychological disposition to act in a certain way (Díez, 2017, p. 136).

As regards beliefs in education, the studies of Pajares (1992) are pioneering, pointing to the early acceptance of beliefs and to a great reluctance to change in adulthood, and, above all, they serve to assess the influence that they have on the decisions made by every teacher on the knowledge they impart, which range from the simple memorising of authoritative sources to the sophistication of critical and creating thinking.

Implicit theories, given their idiosyncratic nature, extend the framework of studies on beliefs, affecting principles, beliefs, goals, expectations, values, and practise models (Mitchell, 1995), and, due to their interdisciplinary study, there are connexions with social representations (Castorina et al., 2005). It is worth highlighting that, in the education context, the implicit theories are reconstructed based on educational knowledge gained through training and work actions (Marrero, 1993) in such a way that, compared with the knowledge representation of the explicit educational theories, the implicit theories entail knowledge attribution, beliefs that the individual pragmatically assumes (Ros-Garrido and Chisvert-Tarazona, 2018, p. 99; Maldonado et al., 2019). As their acceptance reveals a semiological relationship between theory and action, where the intentional perspective is prioritised over linear consistency, it is important to consider the holistic role of teachers, who work not under instruction, but rather as creators of a sense of unity (Zabalza, 1987, p. 115):

teachers do not usually act with rigid and standard systems, but rather they interpret situations, create overall views on the indicators or clues observed in the classroom and act accordingly. It is not, therefore, the case of teachers in a space of certainty and automatic connexions between thought and actions, but rather one of teachers in a context of permanent hypotheticality (“given the situation, I think the best thing I can do is…”) [*sic*].

As such, the scientific consideration on the thinking of teachers in their intellectual intent and complexity allows us to delve into the reason for being of educational processes and in the “re-conceptualisation of educational research with and based on teachers” (Jiménez-Llanos and Feliciano-García, 2006, p. 113). Therefore, it is important to consider the psychological determinants (implicit theories, values, beliefs) and environmental determinants (resources, external situations, administrative limitations) (Fandiño, 2007), as well as the difficulties involved in this study, particularly that relating to the verbalisation of their professional thinking due to two reason: firstly, teachers tend not to communicate it in a reflective manner, but rather through intuition; and, secondly, their ideas and actions often respond to pragmatic and sentimental occurrences, which are difficult to compare with logical-causal categories. Questionnaires are useful instruments for measuring implicit beliefs and theories (Schommer-Aikins, 2004). Specifically, Likert scale questionnaires with multiple choice answers allow the key areas on the subject in question to be covered (Serrano, 2010, p. 274), provided the constructs and dimensions for their matrix are suitable for the assessment purpose sought (Vizcaíno et al., 2015).

### The Implicit Theories of Teachers on the Argumentative Commentary of Multimodal Texts

Addressing the analysis of the implicit theories of teachers on the argumentative commentary of multimodal texts focuses research into what Grossman (1990) understands as “content knowledge” (substantively specialised in a subject matter) and “pedagogical content knowledge” (pedagogical knowledge on the teaching of the specialised subject matter). In this regard, considering their background and current status of this scientific issue is of interest.

Inspired by pioneering studies on the analysis of teacher thinking centred on the teaching of language (Pearson and Stephens, 1994; Woods, 1996; Borg, 2003; White and Bruning, 2005), Spanish studies have been conducted over the last two decades that analyse the interaction of beliefs, assumptions and knowledge as to such regard (Cambra et al., 2000; Ballesteros et al., 2001; Munita, 2013). As for teacher thinking centred on the teaching of Spanish, many more studies have been conducted on Spanish as a native language than on Spanish as a foreign language. There is a thriving flow of research in Spain and Latin America on the thinking of teachers with regard to academic writing (Carlino, 2008; Ortiz et al., 2009; Martins, 2012, 2014; Capomagi, 2013; Castell and Mateos, 2015; Giraldo, 2015; Flores, 2018; Bigi et al., 2019; Cordero and Carlino, 2019; Gordillo, 2019) and on reading literacy and comprehension of academic texts (Makuc, 2008; Suárez, 2015; Mojarro-Delgadillo and Alvarado-Nando, 2021), where diverse implicit theories are usually used: the linear or decoding theory (Laberge and Samuels, 1994), the generative cognition theory (Chomsky, 1974), the interactive or procedural theory (van Dijk and Kintsch, 1983; Rumelhart, 1997); the transactional theory (Goodman, 1994; Rosenblatt, 1996; Mendoza, 2001).

On the other hand, with regard to analytical studies on teacher thinking relating to Spanish as a foreign language, there are only a few publications in different countries (Minervini, 2004; Usó, 2012; Almeda, 2014; Santos and Alexopoulou, 2014; Zhiying, 2018; Domínguez et al., 2019). More specifically, there is an extreme lack of studies that regard beliefs relating to the teaching of argumentation (Martínez, 2018) and there is no known record of studies on the exploration of teacher thinking related to the argumentative commentary of multimodal texts, except those conducted by the research team currently co-led by the authors of this article.

Therefore, the scientific initiative on which this article provides knowledge, opens up a new line of research on an international level. To date, in terms of the paradigm of studying teacher thinking, only one analysis has been published on the teaching needs of professional training in the teaching of argumentation in text commentaries (de Vicente-Yagüe et al., 2019), as well as an exploration of academic teaching customs on the suitable methodology for undertaking informal argumentation in text commentaries that affect the textual typologies, negotiations with students, the procedural sequence of oral and written tasks, and the revision and assessment strategies of said commentaries (Caro et al., 2018).

The exploratory research set out in this document is consistent with a dialogic model of text commentary in its rhetoric architecture sustained over the logical course of informal argumentation (Caro and González, 2018), given that the latter is conceived within a multimodal aspect, both generic and digital, that is inseparable from the functioning of the discourse and that is evident in its linguistic modalisation and is illustrative of any kind (Amossy, 2008, p. 12). Such model starts by looking into the epistemic culture of two inherent activities of the commentator, the interpretation of texts and intertexts, and the formulation of ideas and the writing of the commentary with the hypothesis-arguments-conclusion sequence. In this regard, it is important to be aware of the problem that may reveal the thinking of teachers based on resolute beliefs and customs that, responding to authoritative and mimetic pedagogical models, are reluctant to grant readers inventive power to generate knowledge. Therefore, using exploratory questionnaires on academic customs, beliefs and demands of Spanish language teachers for such purpose, prior validation and analysis of their internal consistency, is a priority objective (Caro et al., 2021).

The professional thinking profile is explained with regard to the classification of teacher responses according to the criteria stipulated relating to the kind of implicit theory and the pedagogical model associated with it. A recurring classification is that of Hargreaves and Goodson (1996, p. 4–19), entailing the following profiles: classical (shared technical culture as organisational self-regulation to provide good customer service), flexible (collaborative culture in communities of professional practise improvement), practical (culture that dignifies practise as a source of knowledge), expanded (connective culture of theory and practise and wide-ranging collective planning), and complex (culture committed to solving problems and uncertainties). However, in view of the innovative expectations with respect to traditional pedagogies on text commentary and argumentation, it is preferable to start from a classificatory model that differentiates with evolutionary clarity the main implicit theories of teaching, such as the one referred to by Marrero (1993, p. 251–255), in these terms schematised by Beltrán (2019, p. 205). See Table 1. We have chosen Marrero's (1993) classificatory model in order to transpose into it the didactic characteristics of text commentaries and argumentation which, guided by our previous research on the subject (Caro and González, 2012, 2015, 2018; Caro, 2015), we specify below in a systematic way in correlation with the type of theory and its corresponding pedagogy. We intend this new classificatory model to serve as a preliminary organiser for the discernment of professional profiles through the teaching responses on the subject (Table 2).

**Table 1:** Generic classification model of implicit teacher theories (Marrero, 1993).

**Type of theory**	**Characteristics**	**Pedagogical model with which it is associated**
Dependent	Teacher-guided and teacher-directed teaching, so that the same pace of learning is maintained for all students; it is thought that, if the teacher does not teach, students are not capable of learning on their own; a distant attitude toward students and a conception of the school outside social and political conflicts are postulated.	Traditional
Productive	Teaching is the pursuit of results and the enhancement of effectiveness in teaching and learning. Teaching by objectives becomes relevant.	Technical
Expressive	It recognises student activity as the core element of the teaching and learning process. Permanent indicators include experimentation, education for life, the number of activities to be carried out and the permanent occupation of students.	Active
Interpretative	Pedagogy centred on students (their needs, resources, and learning processes) and an interpretative attitude (search for more or less formalised explanations of teaching practises) coincide. It stresses the importance of processes over outcomes and emphasises the communicative aspects of teaching.	Constructivist
Emancipatory	It has a strong moral and political character in a broad sense. The concern for the contextual legitimisation of certain objectives and contents of teaching, the link between teaching practises and the political-social framework of the actions of students and teachers accentuate the critical character and the corresponding emancipatory intentionality.	Crítico

**Table 2:** Classification of implicit teacher theories (Caro et al., 2021).

**Type of theory**	**Characteristics**	**Pedagogical model with which it is associated**
Dependent	Logocentric teaching based on the transmission of knowledge from a neoclassical positivist paradigm: (1) It conceives text commentary as an individual representation of the meaning of the text according to the author's ideas. (2) It attributes personal critical argumentation to few genres, but not to commentary. (3) It gives authoritarianism to teachers: their monologue instructs the contents without classroom discussion and without attending to diversity; they make a pyramidal selection of texts (canon); they evaluate the performance of students based on a controlled pattern of concepts and behaviours. (4) It affords passivity to students: reproducing information; practises entail the application of theory. (5) Conservatism (presumed school neutrality). (6) It rejects Information and Communication Technologies (hereinafter, “ICTs”).	Traditional
Productive	Functional teaching for training effectiveness from a positivist paradigm of technical rationality: (1) It conceives text commentary as an individual reconstruction of the meaning of a specialised text and of the author's ideas. (2) It attributes personal critical argumentation to few genres, but not to commentary. (3) Teachers as facilitators of the contents to achieve the objectives: they make a pyramidal selection of texts (canon) and evaluates the students' results from their programmed expert disciplinary control of concepts and skills. (4) Students as consumers of knowledge models; their practises are of a behaviourist nature in terms of theory application. (5) Conservatism (presumed school neutrality). (6) The use of ICTs enhances information.	Technical
Expressive	Spontaneous teaching that promotes learning for life from a humanistic experiential paradigm: (1) It conceives text commentary as an individual reconstruction of the meaning of the text and of the author's ideas, based on which they give a personal opinion. (2) It attributes personal critical argumentation to few genres, including the commentary. (3) Teachers as facilitators of content learning through activities: they take into account the students' tastes in the textual selection (educational canon) and evaluates their performance according to the corresponding experiences. (4) Students as the centre of the teaching-learning process based on the meaningful motivation of the actions. (5) Spontaneous dialogue (no promotion of critical ideology). (6) The use of ICTs enhances feedback.	Active
Interpretative	Cognitive teaching that meets the needs, resources and learning processes from a humanistic interpretative-symbolic paradigm: (1) It conceives text commentary as an individual reconstruction of the meaning of the text (hypertext) from the author's perspective or thesis and as a construction of the critical sense of the commentator through argued hypotheses. (2) The personal critical argumentation is multimodal (including the commentary). (3) Reflective teachers: critical action-research in the teaching-learning processes: it takes into account students' tastes in textual selection (educational canon) and evaluates their procedural performance according to the competences demonstrated in the tasks; interprets teaching practises. (4) Reflective students: the centre of the competence-based teaching-learning process, constructing and self-assessing in a procedural way their tasks. (5) Critical dialogue (ideological perspective). (6) The multimodal use of ICTs enhances feedback.	Constructivist
Emancipatory	Democratic teaching that meets the needs, resources and learning processes based on and organised under a critical emancipatory paradigm: (1) It conceives text commentary as an individual or collective reconstruction of the contextual meaning of the text (hypertext) from the author's perspective or thesis and as a construction of the critical sense of the commentator through argued hypotheses. (2) The personal critical argumentation is multimodal (including the commentary). (3) Teachers as intellectual transformers committed to the sustainable development of the community: critical and meta-reflexive research-action in the teaching-learning processes: they take into account the tastes and ingenuity of the students in the textual selection (educational canon) and in the didactic proposals for social improvement; they evaluate the students' procedural performance according to the competences demonstrated in the contextualised performance of the individual and collaborative tasks. (4) Students as autonomous, responsible and transformative people committed to the sustainable development of the community: the centre of the teaching-learning process based on competences; they learn theory for practical problem solving; construct personal theories from their own research and reading reflection; generate knowledge and experience social empowerment; undertake a self-evaluation in a procedural manner on their performance in the key and global competence framework. (5) Innovative critical dialogue (transformative ideological perspective on equity and diversity). (6) Multimodal communication with ICTs enhances social emancipation.	Crítico

Alongside the implicit theories that are clarified regarding the teaching of argumentative textual commentary, it would be appropriate to critically consider the trends that currently mark its teaching ethos, as teachers are in a transition between the parameters in which they were trained and the need to train for the challenges that the current situation poses. As such, just as a few decades ago, the idea of autonomous professionals who chose the most appropriate methods for their students evolved toward that of associated professionals who carry out their professional development collaboratively to face challenges and uncertainties with daily work in learning communities; in the Knowledge Society, we are moving toward a “post-professional age” where inter-institutional permeation is growing through digital communication and the commercialisation of education is accentuated with client-like relationships that, unfortunately, devalue teachers by denying them their autonomy (Montero and Gewerc, 2018) and that objectify their expectations of competence innovation in consumer banners (Caro, 2017). For this reason, the need to focus teacher thinking on authentic and multicultural communicative expectations (Dorfsman, 2018) that differ from the neo-conductive market impostures, and that advance with emancipatory theories of a critical pedagogical model, is currently gaining scientific momentum.

In the current context, the relevance of online educators and the need to promote the development of their digital competence has increased, as recognised by the TALIS 2018 report (OECD, 2019, p. 12). Educational institutions should approach this challenge with a community commitment that does not reduce it to mere instrumentalist executive work according to neo-behaviourist taxonomic competencies. In this sense, we consider the opportunity to work on heuristic argumentation in authentic situations of shared learning to activate the hypothetical thinking of teacher action-research with the strategic use of digital media (Caro, 2018). Furthermore, we undertake the line of research in the argumentative commentary of multimodal texts where all students, in singular or shared leadership, can expand their critical interpretation and their emancipatory hypotheses. This line has a bearing on a key issue for such pedagogical renewal, as text commentary as an academic discursive genre has been one of the bastions on which teaching based on the transmission of knowledge has survived (Bordieu, 1989, p. 28) by reducing it to dissertation obedient to the principle of authority and the ideological control it entails (Foucault, 1973). In fact, traditional pedagogical models with this theoretical profile, replicated in textbooks, are still applied in schools today (López, 2008; Lluch and Serrano, 2016; Rodríguez-Martínez, 2016).

Therefore, the study of implicit teacher theories on the teaching of argumentative commentary of multimodal texts has to start from an enquiry into their epistemological beliefs about argumentation and text commentary, the assumptions of which are not usually made explicit in teaching practise nor in the teaching models that support them, although they are fundamental to discern two psychological models of understanding the production of knowledge: the mimetic model of “stating” knowledge and the creative model of “transforming” knowledge (Scardamalia and Bereiter, 1992).

Likewise, studying their professional expectations about the use of ICTs in the teaching of argumentative commentary on multimodal texts will allow us to gather valuable information regarding the teachers' vision of technology, allowing several aspects to be contrasted: one is whether they replicate the utopian theses disseminated by educational institutions (neutral and controllable tools that procure prosperity) or, conversely, whether they denounce them in their dystopia (corrupt force that will destroy humanity); the other is whether they possess implicit emancipatory theories in this respect, according to the characteristics pointed out by Castañeda et al. (2018, p. 13) on the emergent network pedagogy as “increased reflective practise,” exercising social engagement with personal learning environments specific to the current technological context.

The outcome of the teacher thinking analysis on this subject will result in proposals on their professionalism (Englund, 1996) or the diagnosis of the quality of their work, taking into account their method and style, and the scientific-technical standards that serve as a framework, all with the aim of reflecting objectively on their improvement through innovative processes.

In line with the aforementioned scientific bases, the objectives of this research are established below:

### General Objective

To interpret the pedagogical profile of Spanish language teachers in their implicit theories on the development of argumentation in the commentary of multimodal texts based on the analysis of their corresponding teaching beliefs.

### Specific Objectives

1. To describe teachers' epistemological convictions on the definition of text commentary (SO1).
2. To identify teachers' preferences on the didactic modalities of text commentary (SO2).
3. To determine teachers' judgments on the generic effect of argumentation (SO3).
4. To explore pragmatic teaching models on the verbal communication of argumentation (SO4).

4.1. To identify argumentative models in expressive activity (SO4.1).
4.2. To recognise argumentative models in the comprehensive activity of text commentary (SO4.2).

5. To analyse teaching assumptions regarding the value that the commentator should place on the wording of the text (SO5).
6. To discover teachers' attributions regarding the argumentative key of commentary (focal point of enquiry/matter of controversy) (SO6).
7. To enquire into teachers' beliefs regarding teaching resources for argumentative text commentary (SO7).

7.1. To explore teachers' judgment on the methodological suitability of textbooks for teaching argumentation in text commentary (SO7.1).
7.2. To explore teachers' expectations of ICTs in argumentative commentary (SO7.2).
7.3. To explore teachers' pedagogical preferences on the design of didactic guides for argumentative text commentary (SO7.3).

8. To discern teachers' implicit theories regarding the argumentative commentary of texts by means of a contrastive analysis of the answers to the exploratory questionnaire according to typological parameters (SO8).

## Materials and Methods

A quantitative methodology based on a non-experimental or *ex post facto* design with semi-structured, closed-ended and survey-questionnaire instruments has been selected. Implicit teacher beliefs or theories about the development of argumentation in text commentary were assessed by means of a Likert-style questionnaire, the design of which was previously inspired by the literature on quantitative research on teaching beliefs (Inguanzo, 2010; Castañeda and Ortiz, 2017; Vizcaíno et al., 2018). For the analysis of the internal consistency of this instrument, the percentage of reliability was measured in the three thematic blocks (teacher customs, beliefs and academic demands) in which the questionnaire was structured, obtaining fairly acceptable results. The analysis of construct validity was also carried out by checking the correlation matrix to determine the degree of variable correlation.

### Sample Population

With a target population made up of teachers of Spanish as a Native Language (hereafter referred to as SNL) and Spanish as a Foreign Language (hereafter referred to as SFL), the teaching staff in the area of Spanish Language and Literature in Spanish-speaking countries at different educational stages and the university teaching staff of SFL in different countries have been established as the sample framework (or study population). The subjects were selected using the non-probabilistic sampling technique, applying the criterion of accessibility. A total of 502 teachers took part in the questionnaire, 390 of which were SNL teachers and 112 SFL teachers.

In order to define the characteristics of the selected population, a series of items related to the socio-demographic and academic data of the teachers surveyed were integrated (Table 3).

**Table 3:** Socio-demographic and professional profile of participants.

Age	Under the age of 20 21–30 31–40 41–50 51–60 61 or more	6 88 138 114 120 28	**Mean:** 43.17
Gender	Male Female Don't know/not answered	142 354 6	
Academic training	Diploma Licentiate degree/degree Master's degree Phd Don't know/not answered	40 200 92 162 8	
Specialised studies undertaken	Philology Language and literature teaching Education or teaching Geography and history, and philosophy Sociology Communication Special education Don't know/not answered	144 62 70 26 4 20 4 172	
Teacher experience	1–5 years 6–10 years 11–15 years 16–20 years 21 or more Don't know/not answered	128 78 54 68 168 6	**Mean:** 15.62
Teaching given	Pre-primary education Primary education Compulsory secondary education (ESO) Further education (*Bachillerato*) University degree Postgraduate degree Does not work on text commentary in class In different educational levels Don't know/not answered	2 60 20 98 92 40 150 18 22	

### Data Collection Instrument

We have developed an *ad hoc* questionnaire consisting of 28 items (see Annex 1), geared toward to SNL and SFL teachers. This is an instrument of open and closed questions digitised electronically using the University of Murcia's survey platform^1^. In terms of its structure, it is made up of two blocks:

#### Block 1: Academic and Socio-Demographic Data

This allows the research to be contextualised and the characteristics of the selected population to be defined. This is made up of a series of items related to the academic data of the teachers surveyed, such as for example, the teaching given, studies undertaken, age, among others. In total, 5 items.

#### Block 2: Teachers' Academic Beliefs

This block collects data related to the academic beliefs of teachers regarding text commentary work in class with variables such as type of analysis and interpretation procedure, dialogue and argumentation or scientific cognition, individual/collective and oral/written modality, use and function of argumentation, beliefs about commentators, teaching manuals, use of ICTs, appropriate procedures for elaborating teaching guides, among others. In total, 23 items.

We have undertaken the reliability analysis and the beginning of the validation process of the data collection instrument, i.e., the analysis of the internal consistency of the questionnaire scale applied in a pilot test (28 items).

## Results

For data processing and analysis, the statistical analysis programme IBM SPSS (version 27) was used. The Cronbach's Alpha coefficient obtained in this block corresponds to 0.8327. Consequently, the reliability of the measurement instrument comprising the set of items, Likert scale 4, with the following levels: “*Strongly disagree,” “Disagree,” “Agree,” “Strongly agree,”* is high.

**Figure 1:**
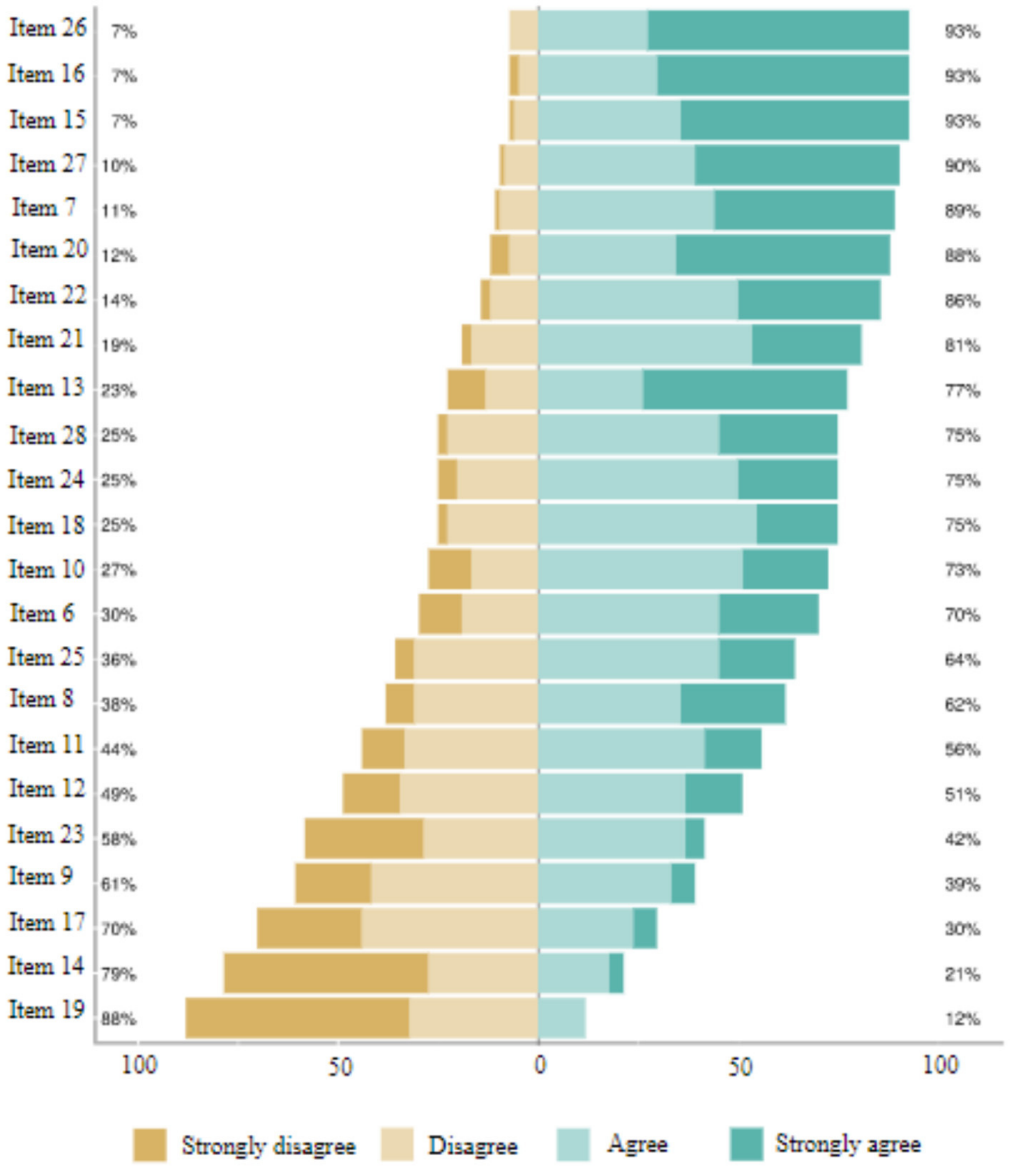
Block 2. Teachers' academic beliefs.

The analysis of grouped relative frequencies has allowed us to explore the distribution and extent of beliefs about argumentative text commentary extracted from the answers given by respondents (Figure 1).

The 23 items corresponding to block 2 of the survey related to the teachers' academic beliefs on argumentative text commentary were as follows:

- Item 6. Text commentary should be first and foremost a procedure of “analysis, interpretation and evaluation of textual data”
- Item 7. Text commentary should be first and foremost a procedure of “dialogue with texts in which a personal position is argued”
- Item 8. Text commentary should be first and foremost a procedure of “scientific cognition that uses language in an interdisciplinary way”
- Item 9. The preferred mode of text commentary should be “individual and oral”
- Item 10. The preferred mode of text commentary should be “individual and written”
- Item 11. The preferred mode of text commentary should be “collective and oral”
- Item 12. The preferred mode of text commentary should be “collective and written”
- Item 13. Argumentation can appear in any type of text
- Item 14. Argumentation can appear only in academic and opinion texts
- Item 15. Argumentation serves to provide a space for discussion between two or more perspectives, ideologies, etc.
- Item 16. Argumentation serves to express a personal or collective position on an issue.
- Item 17. The argumentation of the text commentary focuses primarily on the recognition of explicit aspects
- Item 18. The argumentation of the text commentary focuses above all on the interpretation of the implicit aspects
- Item 19. In text commentary, “what is stated” in the text is presupposed as something unquestionable
- Item 20. Text commentary presupposes “what is stated” in the text as subjective and subject to critical review
- Item 21. The commentator makes incognito enquiries in order to propose a solution and to argue their defence
- Item 22. The commentator chooses controversial issues in order to argue, dispute, deliberate and engage in dialogue with arguments
- Item 23. The textbooks provide teaching material in line with the teaching methodology I consider suitable for argumentation in text commentary
- Item 24. The use of ICTs could improve argumentative skills in text commentary
- Item 25. Focusing the analysis of the text on the understanding of the literal and implicit contents of the author's intention
- Item 26. Contrasting the author's intention with the commentator's perspective in order to promote critical thinking
- Item 27. Providing guidance to the commentator on how to organise the sections and the writing of the argumentative commentary
- Item 28. Giving commentators the freedom to use their critical sense with their own contextualised logic and style.

In order to identify teachers' profiles according to their implicit theories, new categorical variables were established by grouping the contiguous values of the variables studied in block 2 of the questionnaire.

**Figure 2:**
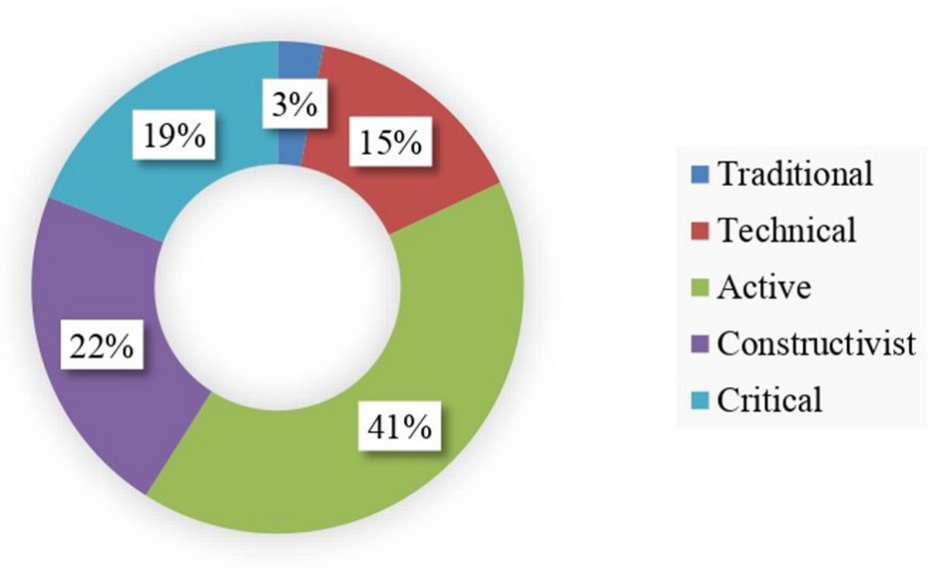
Teacher academic belief model.

From the resulting Gaussian bell, five categories were obtained which described the generic model of classification of teachers' implicit theories on the teaching of text commentary and argumentation described above (see Figure 2). The descriptive statistical analysis showed the distribution of these categories according to the percentages obtained. It became clear that a minority of respondents (2.8% declared themselves as traditional and 15.1% technical) positioned themselves within the parameters that define a conservative, teacher-centred teaching model, focused on the transmission and reproduction of the knowledge given. In contrast, the highest percentage of participants (41.4%) in the study was concentrated in the expressive-active model, based on spontaneous teaching that promotes learning for life from a humanistic experiential paradigm (Figure 2). This may be related to their usual practises of teaching programming and designing classroom projects, where the possibility of specifying the active methodologies for competence training required by the Bologna process is often resolved with simple expressions such as “active and participatory methodology” without specifying the specific systems of task- and project-based learning. This also shows the scarce scientific-academic impact that institutional proposals for initial and ongoing teacher training focused on educational methodologies have on teachers' beliefs, as they do not use them unless they change their habit of relying on the use of manuals for teaching to design projects that favour constructivist and emancipatory learning. It may also be related to the fact that their licentiate degree or degree training in Hispanic Philology and related subjects does not tend to focus on issues of linguistics applied to education.

As for the comparative analysis of the epistemological and didactic convictions linked to the commentary of argumentative texts between teachers of Spanish as a native language (390 respondents) and those of Spanish as a foreign language (112 participants), the only relevant difference was found in the categories related to the constructivist and critical pedagogical model. It was the SFL teachers who registered a greater awareness of democratic teaching based on the critical emancipatory paradigm. This may be related to the methodological parameters established by the Council of Europe in the Common European Framework of Reference for Languages (CEFR) and, specifically, in the Curricular Plan of the Instituto Cervantes, a transcript of the CEFR, for the teaching of Spanish in the world, whose methodological models of study are used with institutional imperative in the teaching of foreign languages. It also introduces SFL teachers to the usual didactic practise that promotes task-based learning and innovative teaching strategies linked to innovative educational materials hosted on institutional training promotion websites.

**Figure 3:**
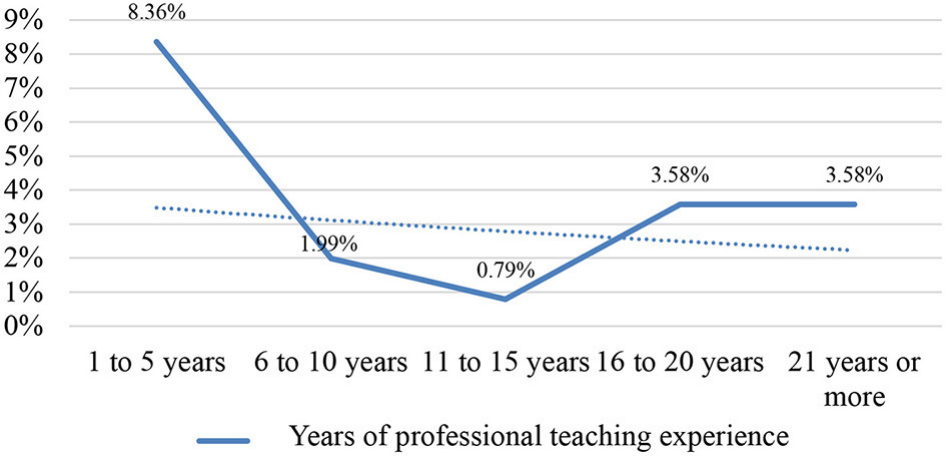
Critical pedagogical model.

**Figure 4:**
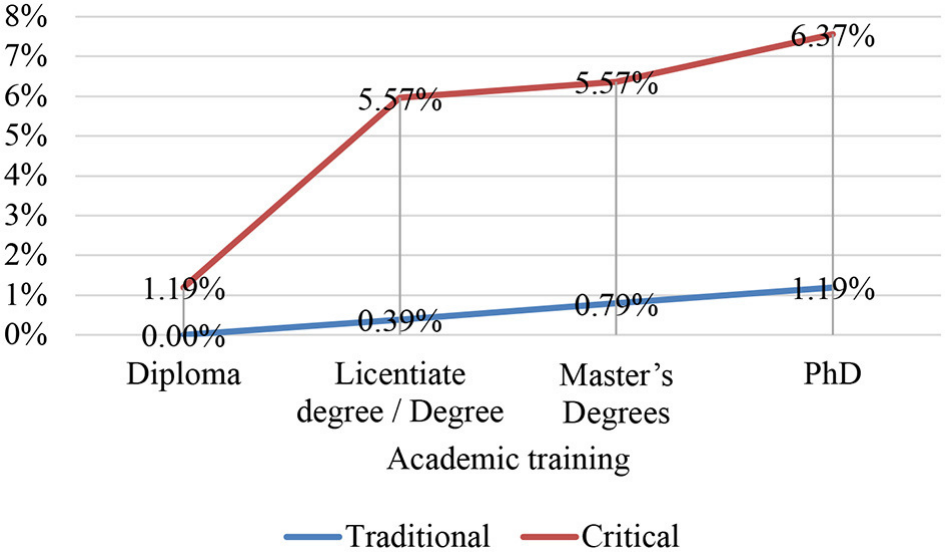
Trends in pedagogical models.

In this sense, the crossover between the variables linked to years of work experience and the model of the academic beliefs of teachers has allowed us to discover that it was the participants with <6 years of teaching experience (in both teaching modalities) who presented a more innovative professional profile, as shown by the exponential trend line in Figures 3, 4. These results may be related to the context of initial training which, for more than a decade, has been offered by the Master's Degree in Teacher Training for Secondary Education in Spain, as, in comparison with the previous professional training system, the Teacher Training Course (*Curso de Aptitud Pedagógica*, CAP), it has improved knowledge of innovative methodologies of a constructivist and emancipatory type by having in its academic programme subjects exclusively for this purpose of pedagogical renovation. This upward innovative dynamic was also observed when looking at the university education of the teachers surveyed: the higher the academic level (Master's and PhD), the greater their commitment was to educational proposals that sought to move away from traditional teaching paradigms. For the same reason, the university teaching staff who teach and guide Master's theses have improved in this respect in their ongoing training and by tutoring educational innovation and research projects, the backbone of which are these methodologies. This aspect was particularly relevant in postgraduate studies linked to the area of Language and Literature Teaching.

Having established the general framework of the models of teachers' academic and pedagogical beliefs, the results related to each of the specific objectives formulated were specified:

### SO1. To Explore Teachers' Epistemological Convictions on the Definition of Text Commentary: Items 6, 7, and 8

In relation to the definition of text commentary, the answers are mainly concentrated on the interpretative aspect of the students (77.29%). Respondents understand that the individual representation of textual meaning according to the author's ideas is not enough, but rather that emphasis should be placed on the student's personal position vis-à-vis the text and, in particular, on the construction of critical meaning through the hypotheses generated, discarded or corroborated during the process of reception. From this perspective, text commentary has been understood as a complex, dialogical, creative, constructive and interactive cognitive process through which the individual participates with full autonomy in different socio-cultural contexts. In this sense, commentators use their cognitive-linguistic skills, activate their analytical thinking to read between the lines, identify and question the underlying ideologies, unravel what is implicit in the statements, argue in a well-founded and contrasted way, etc., through a dialogue between the appellative structure of the text, the author's intentions and the receiver's knowledge of the world and life experiences. This advanced conviction that corresponds to the interpretative theory of reading and its responsive writing in the commentary is consistent with a constructivist pedagogical model that has been consolidated as a belief based on the dialogical conception of communicative competence in the construction of knowledge that teachers of different educational stages have been progressively assimilating in their teaching work and particularly since the LOE (Spanish organic law of education) came into force 15 years ago.

### SO2. To Explore Teachers' Preferences on the Didactic Modalities of Text Commentary: Items 9, 10, 11, and 12

**Figure 5:**
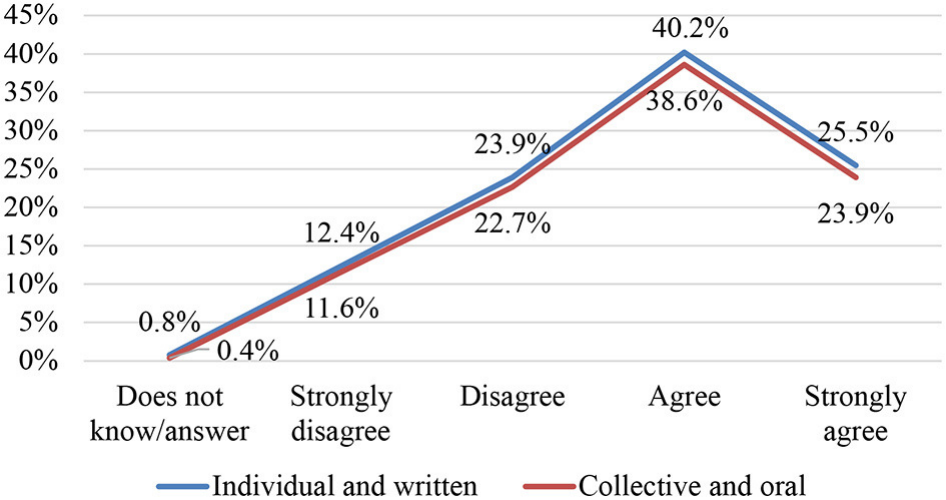
Individual and written text commentary—collective and oral.

The answers given by the participants to the questions related to the oral or written aspect and to the individual or collective nature of the text commentary, made it possible to identify their beliefs in this regard. The identical behaviour of the percentages represented showed two very clear preferences according to the sequential process of the argumentative text and its teaching purpose (Figure 5):

1. 65.7% of those surveyed opted for written text commentary when it came to individually developing the cognitive and linguistic-textual skills and abilities linked to argumentative competence. From this perspective, they have revealed, on the one hand, to possess a graph-centric image by understanding that the written discourse genre favoured a greater degree of formality, planning and critical distancing; and, on the other, didactically perceiving the text commentary as the final product produced and not as a complex constructive process in which interpretative mechanisms come into play, contrasting opinions, as well as cognitive and metacognitive skills associated with analytical thinking and, in general, argumentative competence. Although in their theoretical prolegomena they declare the action of commenting on texts from a dialogical and constructivist approach in accordance with the legal expectations of the twenty-first century curriculum, specific teaching decisions emerge in their teaching practise that contradict this ideal by preferring the written mode of commentary over the oral mode for reasons inherent to implicit theories dependent on the traditional pedagogical model that promotes writing as a product, not as a process. Such a dominant response thus proves the stubborn persistence of the so-called “hidden curriculum” in teachers' implicit theories that disables the proper management of competence-based education.
2. A significant proportion of respondents recognised that textual interpretation by each student should be accompanied by communicative exchange processes in which different points of view are contrasted in order to construct and share a more complex and plural reading (or *archi-reading*) within the interpretative community of the classroom. The emphasis was therefore on transactional exchanges to expose, discuss and reformulate perspectives, ideological positions or simply hermeneutical tasks.

As such, their explicit theories once again clash with their implicit theories, while recognising the benefit of working on the interpretation of texts in communicative acts with group interaction, the responsibilities they grant to text commentary remain individualistic and silent.

### SO3. To Explore Teachers' Judgments on the Generic Effect of Argumentation: Items 13 and 14

Although it is true that a third of the participants admitted that argumentation belongs exclusively to academic and opinion texts, a judgment that reduces the intellectual scope to the genres traditionally recognised as argumentative due to their verbal explanations (stating and defending author's theses), it has been observed that the majority response corresponds to the conviction that it could be found in any discursive genre or textual modality. Such a belief opens the way to the possibility of the emancipatory exercise of argumentation in class, given that it directly affects both the processes of interpretative reading attentive to the implications of the commented text and the criteria for the selection of texts for argumentative commentary, and its predisposition to offer an environment rich in written, oral and multimodal material. Getting students used to dealing with the greatest variety of texts associated with the different contexts of social and cultural life (personal, public, educational, and professional) facilitates specific and different ways of cooperating with the text and of constructing their personal critical point of view in their reading reception writing through commentary, which is no longer an explanatory gloss on the text but an expansion of its meaning in the horizon of each reader's expectations.

With regard to the analysis of the variable of argumentative expression, the teaching judgment prefers to grant argumentation a pragma-dialectical communicative purpose, as it understands that its discursive utility gives rise to spaces for discussion and contrast between different points of view, ideological convictions and beliefs. Therefore, teachers should promote the transformation of the classroom into a space for social interaction between peers, where dialogue after reading is encouraged as a way of:

- Expressing and respecting personal and collective opinion on a given topic.
- Exchanging views, so that no single interpretation is forced on others.
- Consensual negotiating the meaning of the text within the class-community.
- Reaching deeper levels of understanding and interpretation based on individual contributions.

However, the prevailing belief of limiting argumentation to a dialectical model implies that it can only exist in productions or verbal receptions where there is confrontation of criteria and decision-making in favour of or against them, thus neglecting those argumentative initiatives of a focal model that enable the shared construction of knowledge and, therefore, curtailing the treatment of textual multimodality. Moreover, it limits critical commentary to polemical verbal acts subject to verdict and neglects its heuristic possibilities for classroom research.

### OE4. To Explore Pragmatic Teaching Models on the Verbal Communication of Argumentation: Items 15, 16, 17, and 18

The majority of the teachers consulted all believe that argumentation is used to express both singular and plural subjective perspectives and, therefore, they accept without reservation its critical purpose, an aspect due to which a personal critical understanding of the exercise of text commentary can also be accepted, which is interesting for both individual and cooperative oral or written expression actions (Tables 4, 5). It can also be noted that, by referring to the term “position,” in their acceptance of the critical sense, they continue to maintain a dialectical approach to their discursive practise aimed at defending ideas in the face of different or adverse positions.

**Table 4:** Item 15. Argumentation as a space for discussion.

		**Frequency**	**Percentage**	**Valid percentage**	**Cumulative percentage**
Valid	Does not know/answer	4	0.8	0.8	0.8
	Strongly disagree	6	1.2	1.2	2.0
	Disagree	42	8.4	8.4	10.4
	Agree	178	35.5	35.5	45.8
	Strongly agree	272	54.2	54.2	100.0
	Total	502	100.0	100.0	

**Table 5:** Item 16. Argumentation serves to express one's position on an issue.

		**Frequency**	**Percentage**	**Valid percentage**	**Cumulative percentage**
Valid	Does not know/answer	4	0.8	0.8	0.8
	Strongly disagree	6	1.2	1.2	2.0
	Disagree	36	7.2	7.2	9.2
	Agree	178	35.5	35.5	44.6
	Strongly agree	278	55.4	55.4	100.0
	Total	502	100.0	100.0	

In relation to the answers given to questions 17 and 18 regarding the variable of argumentative comprehension, it should be noted that, based on pragmatic assumptions, the concept of implicature was developed due to its essential value in the argumentative processes of text commentary. The recognition of information presented by the sender in a non-explicit way becomes the focus of argumentation in text commentary. This is the perception of the teachers surveyed at least: almost 75% of respondents stated that they agreed or strongly agreed with this statement. This teaching conviction justifies the design of contextualised argumentative practises where students are trained in strategies to identify the implicit and select relevant information in oral and written texts.

However, it should be noted that a significant percentage of participants (33.9%) indicated that recognising what is explicit should be the focus of argumentation. Most are teachers with professional experience of between 1 and 5 years. It becomes clear, in any case, that argumentative discourse consists of an inferential process based on investigating the relationship between what is implicit and explicit in the text.

### SO5. To Explore Teaching Assumptions Regarding the Value That the Commentator Should Place on the Wording of the Text: Items 19 and 20

**Figure 6:**
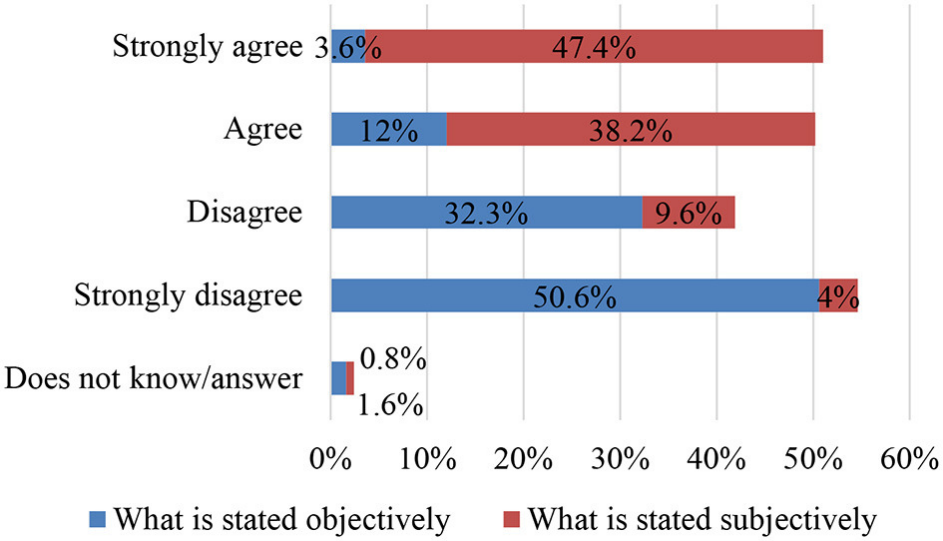
What is stated objectively—what is stated subjectively.

With regard to the analysis of the value of the wording of the text, the teachers surveyed assume that, in text commentary, students should not accept the postulates, premises, opinions or ideological positioning of the author without questioning such. What is explicitly stated in the text needs to be checked and corroborated with other opinions, theories or reasoning. Thus, over 82% of the participants indicate that they disagree with the fact that what is stated in the text should be considered as something indisputable, rather it should be considered as something subjective that is subject to constant critical review (Figure 6). Such a consideration serves to empower the interpreting reader's horizon of expectations in the classroom when establishing a dialogue *between peers*—between two subjective perspectives—with the text, which shows a major advance with respect to the traditional profile of the commentator whose mission is to extract only the authorial sense of the text, as fostering the interpreting reader's power of judgment is very beneficial for leading the way to the implicit emancipatory theory incited by the genuinely democratic practise of commentary in the classroom.

### SO6. To Explore Teachers' Attributes Regarding the Argumentative Key of Commentary (Focal Point of Enquiry/Matter of Controversy): Items 21 and 22

**Figure 7:**
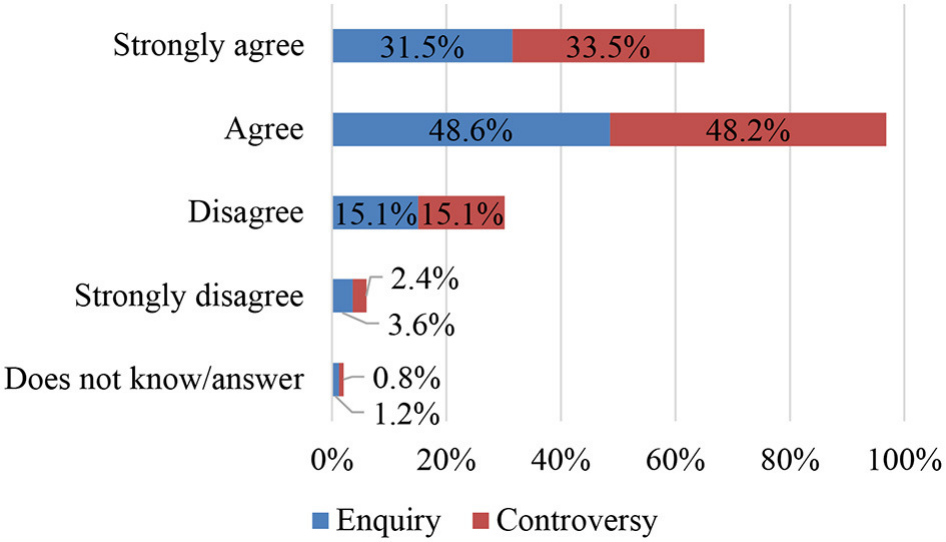
Enquiry—controversy.

In line with the answers given in question 15, teachers' implicit theories tend to attribute to argumentative commentary the defence of a dialectical position which justifies the text and the commentary as a matter of controversy: the purpose of argumentation is to convince interlocutors to adopt a particular point of view or specific opinions. To do so, the starting point must be a communicative situation in which opposing positions are discussed. Based on this belief in controversy as a principle of dialogue, the study participants (over 81%) emphasise the students' selection of controversial issues in relation to which they can debate and deliberate by means of arguments to defend their own theories and refute those of others (Figure 7).

It is incongruous that, with this being their belief regarding the argumentative key of commentary, they attribute preferences of a similar percentage to the key corresponding to the focal point of enquiry, since their fundamental communicative strategy is different to the dialectic one, as they are reasoned commentaries that are contributed in a collective heuristic paper for the shared construction of valid knowledge for the community, thus their nature is predominantly synergic and includes dialectic arguments with fewer resources to reinforce the conviction of their reasoning. It may be the case that teachers have not grasped this distinction of argumentative keys for commenting, as dialectics prevails in legal situations closed by an excluding veridiction and the enquiry key in research situations open to the substantiated postulation of hypotheses as theories that enable problems to be solved by generating knowledge through investigation into unknowns. It is also presumed that in their educational teaching beliefs, there are still no clichés related to research that would allow them to perceive this discernment, nor is there a consolidated line of research on this subject so as to be able to establish them in their initial and ongoing training experience, as research methodology is recent knowledge in their professional training.

It is possible that in the implicit theories through which the teachers have given this answer about the “focal point of enquiry,” it is plausible to argue that in order for the persuasive intention to be effective in convincing the other person of one's own position, it is necessary to activate strategies that encourage solutions to the problem posed through building arguments centred on the cause, through the pursuit of univocity or semantic precision in the definition of the concepts presented, through the use of authoritative quotations, analogy, etc. In this sense, 80% of the sample attributed an essential value to the process of enquiry that the commentator must carry out in order to propose solutions and argue their theory.

### SO7. To Explore Teachers' Beliefs Regarding Teaching Resources for Argumentative Text Commentary: Items 23 and 24

63.4% of teachers consider that textbooks do not provide teaching material in line with the teaching methodology suitable for argumentation in text commentary. One of the major challenges, according to the results obtained, lies mainly in the development of a rich, varied and available repository for the development of argumentative competence and critical reading.

**Figure 8:**
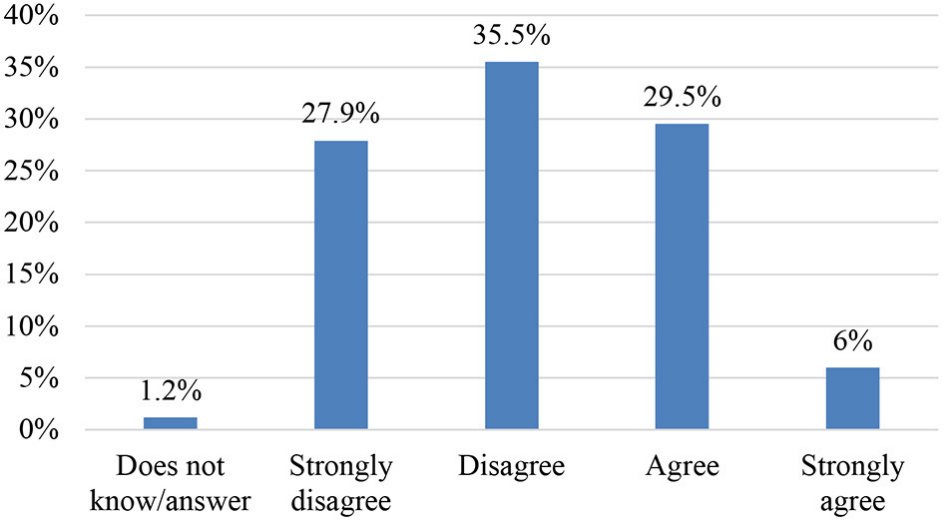
Item 23. Teaching materials in line with the teaching methodology.

**Figure 9:**
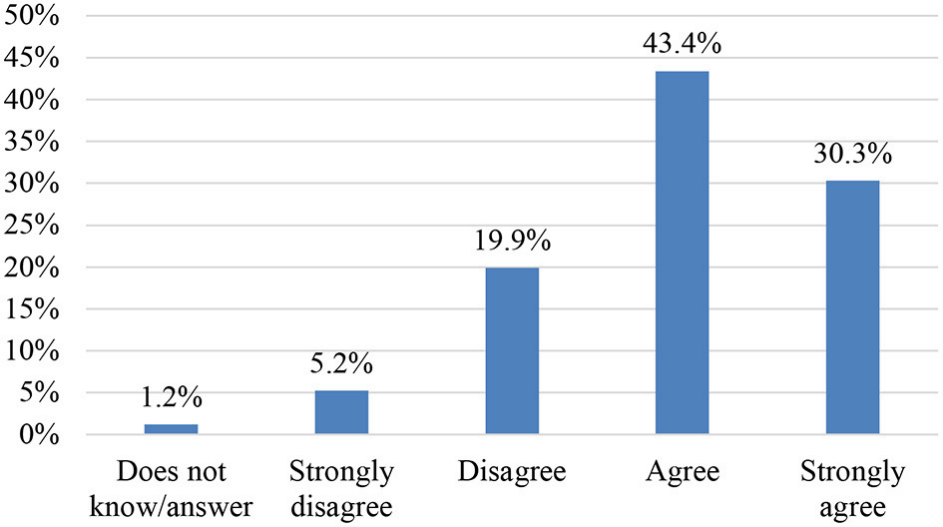
Item 24. The use of ICTs could improve argumentative skills.

In relation to the use of ICT, the majority of respondents assign an important role to ICT regarding improving argumentative skills in text commentaries (Figures 8, 9).

The increasing use of hypertext—the electronic arrangement of networked structures made up of blocks of content interconnected by links, should be taken into account. In this connexion, the links take on the leading role, as they are responsible for allowing the user to move freely through the text, planning or choosing their own route. With a simple click, the reader enters a new textual space and becomes an active part of the argumentative creation process. These possibilities, in many cases, enable the receiver to not only make decisions about their reading project and the construction of their point of view, but also to modify the nature of what is written, manipulate content, contribute documents, transform the discourse, etc. From this perspective, hypertext leads to a multidimensional form of writing and, essentially, a more open and interactive type of hyperlinear reading.

### SO8. To Discern Teachers' Implicit Theories Regarding the Argumentative Commentary of Texts by Means of a Contrastive Analysis of the Answers to the Exploratory Questionnaire According to Typological Parameters: Items 25, 26, 27, and 28

With regard to effective procedures for the teaching of argumentative text commentary, it is noted that the participants tend to strongly agree (90.9%) with the fact that, when designing a teaching guide for effective text commentary, priority should be given to the cognitive processes involved in the contrast between the author's intention and the commentator's perspective in order to promote critical thinking. It is also the case that a high percentage of participants indicate (67.7%) that text analysis should focus on understanding the author's literal and implicit content.

The contrastive analysis of the responses given to questions 25 and 26 reveals incongruence in the options assessed, as it shows that two very different implicit teaching theories are positively regarded with very high and similar percentages: question no. 25 (percentage of responses: 50.60% agree, 17.13% strongly agree) focuses the text commentary guide on the comprehensive analysis of authorial intent and, therefore, corresponds to the type of productive implicit theory associated with a technical pedagogical model for teachers that promotes the consumption of knowledge without giving the commentator the opportunity for critical expression. On the other hand, question 26 (percentage of responses: 31.8% agree and 58.96% strongly agree) focuses this guide on the cognitive processes involved in the contrast between the author's intentional sense and the reading sense that the commentator believes it has in order to promote critical thinking, which corresponds to the types of implicit interpretive and emancipatory theories associated with the constructivist and critical pedagogical models, respectively. Therefore, as there are quite a number of teachers who equally value both issues of very different logic, their attributes reveal a deficit of epistemological discernment which may be due to confused or ambiguous beliefs about the practise of text commentary because they want to balance the functional customs of the profession (which tend to focus only on commenting on the author's arguments) with the innovative expectations of the current pedagogical renewal (which tend to focus on the dialogue between the author's and the reader's arguments).

**Figure 10:**
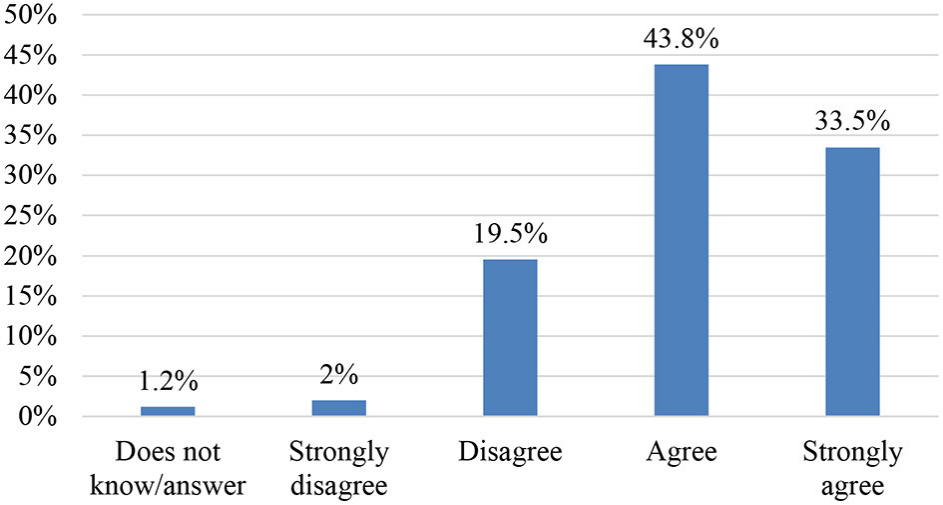
Item 28. Giving the commentator the freedom to design an argumentation guide.

Moreover, regarding the effective procedure for the discursive organisation of the argumentative commentary, 83.27% of teachers prefer the argumentation guide to contain guidelines so that students learn to develop it in their sections and writing appropriately (question 27), and 77.29% of teachers choose to give freedom to the logical, stylistic and contextualised expression of their personal critical sense (question 28). See Figure 10.

Thus, once again, an overlap in the responses emerges in the also positive assessment, with very high percentages for two very different issues: giving guidelines or giving freedom when commenting by giving arguments. It would be appropriate for those who agree with question 27 to disagree with question 28 and vice versa. Nevertheless, the answers given, ranging from “agree” to “strongly agree,” show that teachers' implicit theories on these issues are ambiguous. The cause possibly lies in the fact that they usually practise their teaching methods without having reflected epistemologically on the matter in order to take defined positions, or perhaps it is due to the fact that the training and teaching materials they use have not facilitated such a reflective process, as there are certainly no scientific publications to date that have studied which of the two procedures indicated (with guidelines or free) is the more effective.

## Discussion and Conclusion

The belief system related to argumentative text commentary influences not only the way teachers make decisions and approach their teaching practises in the classroom, but also students' academic performance (Trigwell and Prosser, 1991; Estévez-Nenninger et al., 2014). Our work contributes relevant information to this debate by understanding that the identification of this framework of thinking encourages awareness of actions aimed at transforming educational practise in order to improve the quality of teaching and learning.

In this sense, one of the most significant results of this research helps to classify and frame teacher profiles according to their implicit theories. These pedagogical models are distributed according to the role given during learning to the different education agents, the value and understanding of argumentative text commentary in the development of critical competence, the way of building knowledge and the implication of teaching methodologies.

The diversified results of the eight areas of the exploratory study conducted on teachers' implicit theories regarding the development of argumentation in the commentary of multimodal texts reveal important teacher training deficiencies on the epistemology that should underpin their teaching processes in this respect in order to firmly and clearly move toward the constructivist and critical pedagogical models recommended by current education laws. This finding is in line with the results of previous research (de Vicente-Yagüe et al., 2019), which point to teachers' need for theoretical models on the didactics of argumentation that support and guide the design and implementation of educational proposals leading to the improvement of students' critical competence.

The exploration of teachers' epistemological convictions regarding the definition of text commentary (SO1) provides advanced assumptions corresponding to the interpretive theory of reading and its receptive writing in commentary. This theoretical explanation is consistent with a constructivist pedagogical model that has been consolidated as a manifest belief based on the dialogical conception of communicative competence in the construction of knowledge that teachers of different educational stages have been progressively assimilating in their teaching work since the LOE (Spanish organic law of education) came into force 15 years ago.

However, in the subsequent exploratory aspects (SO2–SO8), the profile of this initial assumption shown as an explicit theory with legal support and professionally accepted by the current institutional context comes into conflict with teachers' other deeper beliefs which reveal the tenacious persistence in their teaching work of the *hidden curriculum* of traditional and technical teaching, as Torres (2005) notes, based on implicit logocentric and functional theories:

- The exploration of teachers' preferences regarding the teaching modes of text commentary (SO2) shows that, although in their theoretical prolegomena they declare the action of commenting on texts from a dialogical and constructivist approach in accordance with the legal expectations of the twenty-first century curriculum, specific teaching decisions emerge in their teaching practise that contradict this ideal by preferring the written mode of commentary over the oral mode for reasons proper to implicit theories dependent on the traditional pedagogical model that promotes writing as a product, not as a process, which prevents the adequate management of competency-based education. Their explicit theories of innovative will again clash with their implicit theories when, while recognising the benefit of working on the interpretation of texts in communicative acts with group interaction, the attributes they concede to text commentary remain individualistic and silent. We agree with previous studies (Aubert et al., 2009; Giménez and Subtil, 2015) which show that teachers continue to identify text commentary as a solitary student activity consisting of unravelling the author's ultimate intention.
- The exploration of teachers' judgments on the generic effect of argumentation (SO3) shows a change in mindset that overcomes the traditional academic confinement of argumentation in the few genres where it is voluntary and explicit, since today's teacher acknowledges that argumentation can appear in any type of text, which implies a predisposition toward the interpretive study of its multimodal implicatures in the different socio-cultural contexts, processes and expressive formats that require the reader's cooperation to establish the meaning of the text. Thanks to the recognition of the multimodal and implicit condition of argumentation—possibly motivated by the open vision of textuality that the intense use of ICTs in the Knowledge Society provides, text commentary can cease to be understood as an explanatory note on the text and become an expansion of its meaning through the hypertextual horizon established by the interpretive and expressive expectations of each reader. This conclusion is reinforced by the analysis of the results of SO7, where the majority of teachers highlight the insufficiency of the teaching materials available to teach argumentation in text commentary, and rely on the competent use of ICT to improve students' argumentative skills due to the real training opportunities relating to strategic, creative and critical maturity provided by multidimensional and open communication in hypertextual dynamics.
- The exploration of pragmatic teaching models on the verbal communication of argumentation (SO4) visualises, in the analysis of argumentative expression, the dialectical approach prevailing in such models with deep cultural and authoritative roots that continue to give prevalence to the legal rhetorical model of argumentation that translates dialogue as the discussion of opposing positions, whose democratic education emphasises respectful speaking times and consensual negotiation of outcomes. Indeed, this vision of the dialogic learning of argumentative expression as a dispute between antithetical positions stems from the strong roots of legal argumentation in academic discursive practises, maintained from Greek classicism to the present day (Plantin, 2005), without any loss, in a communicational facticity approach (van Eemeren and Grootendorst, 1984) which has been included in language teaching studies for decades (Camps, 1995; Cros, 2003).

We regret that, due to this belief where dialectics monopolises the notion of dialogue, the construction of knowledge does not progress beyond dissension or consensus, toward other heuristic and emancipatory spaces of coexistence and science, as these would be possible if it were understood that dialogic communication can take place by cooperating in focus groups with shared synergies where debate is a minor factor and not the centre of the argumentative activity essential to undertake, develop and finalise common projects with a plausible sense. Previous papers insist on the need to deconstruct teaching habits that are opposed to more democratic and dialogical channels of participation in classroom dynamics (Caro et al., 2018). We believe that it is necessary to generate educational research knowledge to tackle this problem, providing teachers with the necessary training to relativise dialectical argumentation in text commentary as one of its possible teaching approaches, as, in addition to commenting on controversial texts whether for or against, personal critical commentary could be used in the classroom to launch heuristic hypotheses, for example, to explain an unknown or to formulate a challenge with convincing arguments. We believe that, in this way, the deliberative argumentation of commentary would leave behind the only area of confrontations where only opinion serves to advance toward the open field of research that opens up and substantiates knowledge.

Moreover, the dialectical approach is consistent with the professional belief in the subjectivity of argumentation for both individual and collective expression and this supports the widespread conviction of the critical practise of text commentary.

With regard to argumentative comprehension, the predominant professional belief regarding the essential value of reading in depth the implicatures of the texts commented on represents another indispensable step forward in their mature convictions to design relevant and well-contextualised argumentative practises, making it clear that quality argumentative discourse requires an inferential process based on enquiries that clarify the implicit meaning and sense elicited from textual explicitness.

- The exploration regarding the value that the commentator should place on the wording of the text (SO5), given that the majority of responses show a tendency to subjectively and critically question it, provides a rationale for pedagogical initiatives in line with the current principles of competency-based education, as such a consideration serves to empower the interpreting reader's horizon of expectations in the classroom when establishing a dialogue *between peers*—between two subjective perspectives—with the text and its author. This shows a major advance with respect to the traditional profile of the commentator whose mission is to extract only the authorial sense of the text, as fostering the interpreting reader's power of judgment is very beneficial for leading the way to the implicit emancipatory theory incited by the genuinely democratic practise of commentary in the classroom.
- The exploration conducted on the attributes regarding the argumentative key of commentary (SO6) focused on two different possibilities: the dialectic key, which prevails in legal situations closed by an excluding veridiction (van Eemeren and Houtlosser, 2002), and the enquiry key, which prevails in investigative situations open to the substantiated postulation of hypotheses enabling problem-solving by generating knowledge from unknowns. The results analysed are consistent with the results of SO4, as once again dialectics predominates as the key to justifying commentary as a discursive space for taking a stance on controversies with arguments defending a certain theory. Nevertheless, as the teachers also rated the key to the focal point of enquiry with a similar percentage, which unfolds when the commentary runs heuristically with synergies for the shared construction of knowledge, one of the following two possibilities can be interpreted: teachers accept both argumentative keys, or they lack epistemological discernment between the two, as there is still no consolidated line of research in the area of Spanish language teaching regarding the key to the focal point of enquiry applied to text commentary that may have been disseminated in initial and ongoing teacher training and, furthermore, research methodology is recent knowledge in their professional training.
- Finally, the discernment of teachers' implicit theories on the argumentative commentary of texts regarding the comprehension and expression strategies that an *ad hoc* teaching guide should offer (SO8) shows their preference for dialogue between the author's theory and the commentator's critical hypothesis. However, this presumed critical emancipation of teachers is not clear due to the epistemological deficit revealed in the analysis of results, as they preferred in high and similar proportions two questions concerning disparate pedagogical models, which could be related to the dissociation that exists between their habits in the classroom (comments made adhering to guidelines and comments on text content and author's arguments) and the prologues of the laws governing the curriculum (dialogical and critical approach; freedom of expression), as well as the shortage of training opportunities and professional publications to reflect on such educational issues in focus groups.

The findings of this study raise additional questions that could open up new lines of work. The identification of teachers' beliefs involves the construction of a training proposal which, based on constructivist and critical pedagogical models, is the independent variable in quantitative experimental research with two treatment and comparison groups.

## Data Availability Statement

The raw data supporting the conclusions of this article will be made available by the authors, without undue reservation.

## Author Contributions

All authors listed have made a substantial, direct and intellectual contribution to the work, and approved it for publication.

## Funding

This research has been derived from the R + D + I Project entitled Epistemic innovation of a model of argumentative comment of multimodal texts in the teaching of Spanish as a mother and foreign language (ref. PGC2018-101457-B-I00), funded by FEDER/Ministry of Science and Innovation - State Research Agency of the Government of Spain.

## Conflict of Interest

The authors declare that the research was conducted in the absence of any commercial or financial relationships that could be construed as a potential conflict of interest.

## Publisher's Note

All claims expressed in this article are solely those of the authors and do not necessarily represent those of their affiliated organizations, or those of the publisher, the editors and the reviewers. Any product that may be evaluated in this article, or claim that may be made by its manufacturer, is not guaranteed or endorsed by the publisher.

## Footnotes

^1^https://encuestas.um.es.
