# Supplementary material for: Implicit Teacher Theories Regarding the Argumentative Commentary of Multimodal Texts in the Teaching of Spanish as a Native and Foreign Language
Source: Front Psychol. 2021 Oct 27;12:749426. doi: 10.3389/fpsyg.2021.749426 (PMC8579127; doi:10.3389/fpsyg.2021.749426)
Supplement: Supplementary file 1 [file Data_Sheet_1.pdf]

1 Annex 1. Questionnaire.

BLOCK 1. ACADEMIC AND SOCIO-DEMOGRAPHIC DATA

1. Which courses and specialisations do you teach?:

2. Age:
3. Gender: [ ] Male [ ] Female
4. Years of teaching experience:
5. Education (school graduate, diploma-holder, degree-holder, PhD-holder) and specialisation:

BLOCK 2. TEACHERS' ACADEMIC BELIEFS

Please give one answer only as to how you feel about the following statements:

Rate them according to the following scale: 1 "Strongly disagree", 2 "Disagree", 3 "Agree", 4 "Strongly agree".

	1	2	3	4
6. Text commentary should be first and foremost a procedure of "analysis, interpretation and evaluation of textual data"				
7. Text commentary should be first and foremost a procedure of "dialogue with texts in which a personal position is argued"				
8. Text commentary should be first and foremost a procedure of "scientific cognition that uses language in an interdisciplinary way"				
9. The preferred mode of text commentary should be "individual and oral"				
10. The preferred mode of text commentary should "individual and written"				
11. The preferred mode of text commentary should be "collective and oral"				
12. The preferred mode of text commentary should be "collective and written"				
13. Argumentation can appear in any type of text				
14. Argumentation can appear only in academic and opinion texts				
15. Argumentation serves to provide a space for discussion between two or more perspectives, ideologies, etc.				
16. Argumentation serves to express a personal or collective position on an issue.				
17. The argumentation of the text commentary focuses primarily on the recognition of explicit aspects				
18. The argumentation of the text commentary focuses above all on the interpretation of the implicit aspects				

19. In text commentary, "what is stated" in the text is presupposed as unquestionable				
20. Text commentary presupposes "what is stated" in the text as subjective and subject to critical review				
21. The commentator makes incognito enquiries in order to propose a solution and to argue their defence				
22. The commentator chooses controversial issues in order to argue, dispute, deliberate and engage in dialogue with arguments				
23. The textbooks provide teaching material in line with the teaching methodology I consider suitable for argumentation in text commentary				
24. The use of ICTs could improve argumentative skills in text commentary				

Consider which procedure would be most effective when designing a teaching guide on text commentary.

Rate them according to the following scale: 1 “Strongly disagree”, 2 “Disagree”, 3 “Agree”, 4 “Strongly agree”.

	1	2	3	4
25. Focusing the analysis of the text on the understanding of the literal and implicit contents of the author's intention				
26. Contrasting the author's intention with the commentator's perspective in order to promote critical thinking				

Consider which procedure would be most effective when designing an argumentation guide on text commentary.

Rate them according to the following scale: 1 Strongly disagree, 2 Disagree, 3 Agree, 4 Strongly agree.

	1	2	3	4
27. Providing guidance to the commentator on how to organise the sections and the writing of the argumentative commentary				
28. Giving commentators the freedom to use their critical sense with their own contextualised logic and style				
